# Mobility-Based Smartphone Digital Phenotypes for Unobtrusively Capturing Everyday Cognition, Mood, and Community Life-Space in Older Adults: Feasibility, Acceptability, and Preliminary Validity Study

**DOI:** 10.2196/59974

**Published:** 2024-11-22

**Authors:** Katherine Hackett, Shiyun Xu, Moira McKniff, Lido Paglia, Ian Barnett, Tania Giovannetti

**Affiliations:** 1 Department of Psychology and Neuroscience Temple University Philadelphia, PA United States; 2 Department of Biostatistics, Epidemiology, and Informatics University of Pennsylvania Philadelphia, PA United States; 3 Information Technology College of Science & Technology Temple University Philadelphia, PA United States

**Keywords:** digital phenotyping, digital biomarkers, monitoring, mHealth, cognition, mobility, life space, depression, location data, Alzheimer disease, aging, mobile phone

## Abstract

**Background:**

Current methods of monitoring cognition in older adults are insufficient to address the growing burden of Alzheimer disease and related dementias (AD/ADRD). New approaches that are sensitive, scalable, objective, and reflective of meaningful functional outcomes are direly needed. Mobility trajectories and geospatial life space patterns reflect many aspects of cognitive and functional integrity and may be useful proxies of age-related cognitive decline.

**Objective:**

We investigated the feasibility, acceptability, and preliminary validity of a 1-month smartphone digital phenotyping protocol to infer everyday cognition, function, and mood in older adults from passively obtained GPS data. We also sought to clarify intrinsic and extrinsic factors associated with mobility phenotypes for consideration in future studies.

**Methods:**

Overall, 37 adults aged between 63 and 85 years with healthy cognition (n=31, 84%), mild cognitive impairment (n=5, 13%), and mild dementia (n=1, 3%) used an open-source smartphone app (mindLAMP) to unobtrusively capture GPS trajectories for 4 weeks. GPS data were processed into interpretable features across categories of activity, inactivity, routine, and location diversity. Monthly average and day-to-day intraindividual variability (IIV) metrics were calculated for each feature to test a priori hypotheses from a neuropsychological framework. Validation measures collected at baseline were compared against monthly GPS features to examine construct validity. Feasibility and acceptability outcomes included retention, comprehension of study procedures, technical difficulties, and satisfaction ratings at debriefing.

**Results:**

All (37/37, 100%) participants completed the 4-week monitoring period without major technical adverse events, 100% (37/37) reported satisfaction with the explanation of study procedures, and 97% (36/37) reported no feelings of discomfort. Participants’ scores on the comprehension of consent quiz were 97% on average and associated with education and race. Technical issues requiring troubleshooting were infrequent, though 41% (15/37) reported battery drain. Moderate to strong correlations (*r*≥0.3) were identified between GPS features and validators. Specifically, individuals with greater activity and more location diversity demonstrated better cognition, less functional impairment, less depression, more community participation, and more geospatial life space on objective and subjective validation measures. Contrary to predictions, greater IIV and less routine in mobility habits were also associated with positive outcomes. Many demographic and technology-related factors were not associated with GPS features; however, income, being a native English speaker, season of study participation, and occupational status were related to GPS features.

**Conclusions:**

Theoretically informed digital phenotypes of mobility are feasibly captured from older adults’ personal smartphones and relate to clinically meaningful measures including cognitive test performance, reported functional decline, mood, and community activity. Future studies should consider the impact of intrinsic and extrinsic factors when interpreting mobility phenotypes. Overall, smartphone digital phenotyping is a promising method to unobtrusively capture relevant risk and resilience factors in the context of aging and AD/ADRD and should continue to be investigated in large, diverse samples.

## Introduction

### Background

Alzheimer disease and related dementias (AD/ADRD) place immense pressure on our health care system. They represent a global issue that is worsening and will be exacerbated by insufficient disease screening methods and a lack of ecologically valid outcome measures for clinical trials [1]. New and innovative approaches for early detection and monitoring are direly needed to address this global crisis. In this paper, we present results from a proof-of-concept study demonstrating the promise of smartphone digital phenotyping to capture clinically relevant risk factors and outcomes in the context of aging and AD/ADRD.

Decades of clinical trial research indicate that early intervention will be critical for effective AD/ADRD treatment [2-5]. Biomarker testing (eg, positron emission tomography neuroimaging and cerebrospinal fluid), traditional neuropsychological evaluation, and clinician- or informant-rated assessments have historically been the gold-standard methods for detection and diagnosis. However, these methods present drawbacks, including limited accessibility (eg, proximity and cost), scalability (ie, logistical constraints for wide-scale clinical implementation), prognostic value (ie, inconsistent correspondence with clinical progression), and ecological validity (ie, poor representation of real-world functioning) [6-10]. Traditional neuropsychological measures are also affected by sociocultural factors, such as educational quality, socioeconomic status, native language status, and acculturation, rendering them inappropriate for the increasingly diverse global population [11-20]. Subtle difficulties in complex everyday tasks signal early decline and are critical to assess; however, standard functional assessments are limited by recall bias, availability of an informant reporter, bias due to attributes of the informant, outdated items, and poor sensitivity [21-27]. Patterns and trajectories of cognitive and functional decline are also extremely heterogeneous and person-specific [28-30] and are thus difficult to assess using a one-time standardized assessment measure. Taken together, health care systems are ill-equipped to screen for early signs of cognitive impairment at scale, as evidenced by recent estimates that 92% of cases of mild cognitive impairment (MCI) remain undiagnosed [31-34]. In addition to poor screening methods, traditional clinical trial outcome measures are not sufficiently meaningful and precise to demonstrate therapeutic benefit at early stages [4,25,35-40]. Thus, a growing priority in the field of AD/ADRD is to develop and implement sensitive and functionally meaningful screening and outcome measures as new therapies are evaluated earlier in the disease course [41].

New digital assessment methods have great potential for efficient, accessible, sensitive, and objective assessment of early cognitive and functional changes reflecting risk for AD/ADRD [6,7,42-46]. Digital tools can capture microlevel behavioral data with increased sensitivity and reduced sample size requirements compared with traditional paper-and-pencil neuropsychological measures and functional scales [47]. Gathering this information at home can address accessibility limitations for those in rural environments or who face hardships traveling to and from a clinical site, and may provide a more reliable and ecologically valid representation of real-world functioning compared to traditional evaluation at a single time point in a highly controlled setting [48-50]. As global technology use and affordability of personal devices continue to rise [51,52], new technologies can potentially address crucial gaps in the scalability and accessibility of current methods and counteract higher rates of missed diagnosis among populations experiencing low socioeconomic status [34,53-57].

Digital phenotyping is one innovative approach that uses the “moment-by-moment quantification of the individual-level human phenotype in situ” based on interactions with technology, including smartphones and smart home devices, to capture social and behavioral data passively, continuously, and with minimal interference [58-60]. It collects high-frequency, fine-grained data reflecting everyday behaviors “in the wild” without relying on user engagement, subjective reports, or burdensome procedures. Preliminary support has been demonstrated in psychiatry studies leveraging a host of sensors (eg, GPS, accelerometer, Wi-Fi or Bluetooth signals, ambient sound and light, app use, call and text message metadata, and keystroke dynamics) and imputed behavioral features (eg, time spent at home, sleep cycles, level of socialization, and routine or anomalies) to predict clinical outcomes, including depression and bipolar disorder symptoms, suicide risk, psychosis relapse, and depression treatment response [61-70]. In the context of neurodegenerative disorders, several sensors—particularly keystroke dynamics and phone or battery use metrics—have shown associations with neuropsychological test performance, diagnostic severity, and even gray matter volume in clinical cohorts, including those with MCI, Alzheimer disease (AD), frontotemporal dementia, and multiple sclerosis [71-77].

Basic questions of feasibility, acceptability, and ethical considerations related to data privacy are important to weigh when considering the highly sensitive nature of digital phenotyping data in vulnerable populations [78-80]. Few studies have proactively addressed these questions in the context of older adults with cognitive decline [65]. Furthermore, many existing studies have used exploratory approaches without a priori hypotheses [81]. As described by Hackett and Giovannetti [7] and Leaning et al [66], some of the many interpretive and logistical challenges of digital phenotyping can be mitigated with conceptual models and clinically informed features to provide context to results and improve reproducibility.

In this paper, we present findings from a proof-of-concept study evaluating a smartphone digital phenotyping protocol to assess cognition, everyday function, and mood in a cohort of older adults with and without cognitive decline. Here, we focus on smartphone-derived GPS data as the digital phenotyping sensor of interest. Our study design and analytic approach were informed by a conceptual framework proposed by Hackett and Giovannetti [7] based on established trends in cognitive neuroscience, neuropsychology, neurology, and computer science literature [7]. The conceptual framework (ie, the Variability in Everyday Behavior [VIBE] model) posits that pathological cognitive decline is accompanied by a reduction in everyday activities, worsening mood, and lower scores on standardized neuropsychological measures. These declining mean-level trends occur alongside increases in intraindividual variability (IIV) on measures of cognition and everyday function as individuals become more inefficient and work to compensate for underlying disease progression [7]. Trends of decreasing *levels* of everyday activity and increasing *variability* in everyday activities may be indexed by passively obtained smartphone data such as GPS trajectories, hence the focus of this study.

### Objectives

The primary aims of this study were twofold: (1) to examine the feasibility and acceptability of a digital phenotyping protocol among older adult smartphone users, and (2) to examine associations between passively obtained GPS movement trajectories collected over a 1-month study period and traditional validated measures of cognition, everyday functioning, mood, and mobility habits collected at baseline. Data were collected using the Learn, Assess, Manage, and Prevent (LAMP) platform, an open-source platform for research and clinical use, via the mindLAMP app [82-84]. Data for the second aim were analyzed and interpreted according to the following a priori hypotheses based on our conceptual framework (ie, the VIBE model), shown in Textbox 1.

**Hypotheses informed by the Variability in Everyday Behavior model for individuals along the continuum from healthy cognition to MCI.**

**Cognition and function**

Average GPS metrics of activity will show a positive linear relation with measures of cognition and function, whereas GPS intraindividual variability (IIV) for all categories will show a negative relation with cognition and function.

**Mood**

Average GPS metrics of activity will show a negative linear relation with depression (ie, greater overall mobility will be associated with less depression), whereas GPS IIV for all categories will show a positive relation with depression (ie, greater mobility variability will be associated with more depression).

**Mobility**

Average GPS metrics of activity will be positively associated with objective and subjective measures of gait speed, life space, and community participation.

The study also included two exploratory aims: (1) to examine relations between GPS features and participant intrinsic and extrinsic factors (eg, sociodemographic and contextual) to inform the selection of covariates or moderating variables in future studies and (2) to explore whether patterns of mobility—rather than absolute amounts of mobility—also relate to validators. Overall, our results provide preliminary support for the feasibility, acceptability, and validity of digital phenotyping in older adults, along with key insights that can be used to inform future studies.

## Methods

### Recruitment

Participants aged >60 years with healthy cognition, diagnoses of MCI, or mild AD were recruited from specialty dementia clinics and the community within the Philadelphia region, beginning in January 2022. Recruitment also involved contacting previous participants of other research studies within our laboratory, consistently attending community outreach events to establish trust and familiarity with our research team, and providing educational presentations on topics related to cognitive health and aging at local community centers. Individuals who expressed interest in participating in our study were contacted by a member of the study team to schedule a study appointment. During the scheduling call, the team member reviewed basic eligibility criteria (eg, age, use of a smartphone, and availability of a study partner), and participants who met criteria were scheduled for an initial session. Inclusion and exclusion criteria were reviewed again in detail at the start of the baseline session to ensure eligibility. General inclusion criteria for all participants were (1) aged ≥60 years, (2) fluent in English, (3) existing smartphone user (iOS or Android; meeting minimum software version compatibility) for at least 1 year before joining the study, (4) Wi-Fi connectivity at home, (5) phone use on a daily basis; (6) no plans to purchase or switch to a new smartphone over the next 4 weeks, and (7) availability of an informant reporter who has knowledge of the participant’s daily functioning. Exclusion criteria were (1) a history of severe psychiatric or nervous system disorders (other than dementia), (2) current metabolic or systemic disorders, (3) severe sensory or motor deficits precluding smartphone use, (4) intellectual disability, and (5) scheduled surgery or major travel over the 4-week study period. Participants with self- or clinician-reported diagnoses of healthy cognition, MCI, or mild AD completed comprehensive neuropsychological testing during a baseline visit (as described in the subsequent sections), and Jak/Bondi neuropsychological actuarial criteria were used to confirm diagnostic group membership [85]. Follow-up consensus diagnosis was used to account for atypical clinical factors that may impact the accuracy of actuarial diagnosis (eg, English as a second language and co-occurring mood or psychiatric concerns).

Study informants were also recruited for each participant to corroborate responses on the self-reported functional decline validation measure, to confirm other data pertinent to clinical history, and to provide assistance with potential technical difficulties—particularly for participants with MCI or dementia. General inclusion and exclusion criteria for all informants included (1) aged ≥18 years; (2) fluent in English; (3) cognitively healthy with no diagnosis of dementia, MCI, or other neurological and psychiatric disorder; (4) available and willing to complete study questionnaires in person, by phone, or by web; (5) having at least weekly contact with the participant; and (6) reports that they are knowledgeable of the participant’s daily functioning and smartphone use.

### Study Procedures

#### Study Timeline

**Figure 1:**
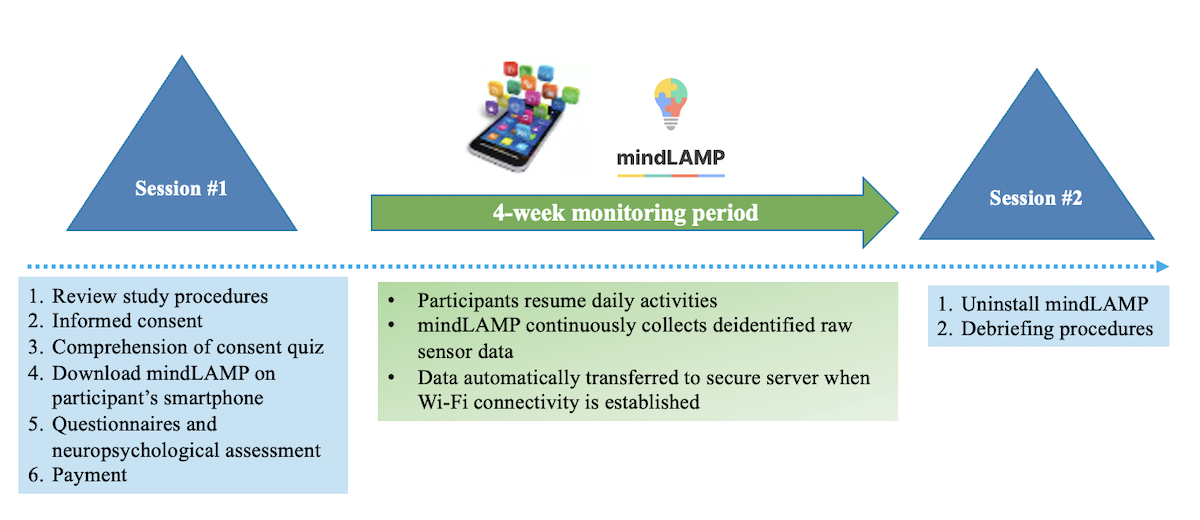
Study timeline.

Participants meeting eligibility criteria were scheduled for an in-person visit (session 1) and were enrolled in the study for approximately 4 weeks. As outlined in Figure 1, participants and informants completed 2 study visits, separated by 4 weeks. Session 1 lasted 2 to 4 hours and included a detailed review of study procedures, informed consent, comprehension of consent quiz, cognitive testing, questionnaires, and configuration of the study app (mindLAMP) on participants’ personal smartphones (ie, downloading mindLAMP, logging in using participant-specific secure credentials, and enabling continuous location services). At the completion of session 1, participants were instructed to resume their daily routines for 4 weeks and were compensated US $50 upfront for their participation. They were asked not to enter low battery or airplane mode and not to log out of the mindLAMP app, which would impact GPS data quality. During the 4-week study period, mindLAMP passively and securely collected GPS data without user engagement. At the end of the study period, participants completed session 2 (in-person or remotely), which consisted of debriefing questionnaires. The mindLAMP app was deleted from participants’ smartphones at session 2, halting data collection.

#### Privacy and Security Safeguards

The LAMP platform was used for the collection of digital phenotyping (GPS) data via the mindLAMP smartphone app, which is available for free on the Apple App Store and Google Play Store. LAMP is an open-source platform for research and clinical use developed by the Division of Digital Psychiatry at Beth Israel Deaconess Medical Center [83]. It uses industry-standard encryption protocols to render information collected from smartphones unidentifiable and transmits data to a secure cloud database via Health Insurance Portability and Accountability Act (HIPAA)–compliant methods whenever Wi-Fi connectivity is established. Participants logged in to mindLAMP via a randomly generated 10-digit user ID (UID) generated within the LAMP platform by the study coordinator, which was only used for the collection of digital phenotyping data. Therefore, no personally identifiable information is associated with the data collected by mindLAMP. Participants’ 6-digit study ID, used for all other clinical data collected within the laboratory at Temple University, is linked to their mindLAMP UID on a university-approved secure research database only (REDCap [Research Electronic Data Capture; Vanderbilt University]).

The aforementioned security and privacy information was thoroughly reviewed with participants during informed consent at session 1. This process included a review of written and visual handouts depicting privacy safeguards, examples of the scope of data collected, and results of other published studies that used mindLAMP [86]. Participants could choose to end study participation and have their study data deleted at any time. After reviewing the consent form, a 10-item comprehension of consent quiz with yes or no response options was administered to ensure participants completely understood the information outlined in the consent form. This quiz covered details including the purpose of mindLAMP (eg, “This study requires downloading the mindLAMP app which collects information from my smartphone sensors”), possible risks or benefits to study participation, such as potential battery drain (eg, “It is possible that I will notice a reduction in my phone’s battery life while mindLAMP is running on my phone”), and data security and encryption methods (eg, “The mindLAMP app uses a secure encryption system called ‘hashing’ to make all information that it collects unidentifiable and untraceable”). Multimedia Appendix 1 presents the complete comprehension of consent quiz. Incorrect items were further reviewed until comprehension was established. These procedures were informed by the Digital Health Checklist and other materials from the Research Center for Optimal Digital Ethics health team, which encourages digital health researchers to proactively identify gaps in the communication of study risks, benefits, and privacy and security details [78,79].

#### Backend Technical Implementation

Data collection and storage were supported by a self-deployed version of the open-source LAMP Platform. A Temple University-approved secure cloud server (1-TB capacity) was purchased and configured with the LAMP application programming interface to enable research participants to connect to the LAMP platform and access the mindLAMP app. Study data were stored in our instance of the mindLAMP database (ie, our copy of the LAMP platform located on a study-specific cloud server) via CouchDB, ensuring a standardized data format consistent with other studies using the LAMP platform [84]. The functionality of the mindLAMP app itself is continually maintained by the team at mindLAMP at the Beth Israel Deaconess Division of Digital Psychiatry. Ongoing security monitoring and backup of study data were maintained by Temple University IT. To monitor unexpected periods of missing data due to potential technical issues (ie, phone powered off, mindLAMP logged out, or permissions reset), we created a code to automate an email alert to the study team (Cronjob) when there were >3 days of missing sensor data. In these instances, the email message included the UID and the corresponding missing sensor, and a member of the study team promptly reached out to the participant to troubleshoot.

### Measures

#### Feasibility and Acceptability

To assess feasibility, we tracked the number of participants who completed the 4-week study period after providing consent and completing session 1 and those who requested to withdraw for any reason. Feasibility was also operationalized by performance on the comprehension of consent quiz, which demonstrates participants’ ability to comprehend complex technical information specific to digital phenotyping studies. To gauge acceptability of the informed consent and overall study procedures, we administered a debriefing survey at session 2 after participants had completed the full study period. Participants were asked to rate their level of satisfaction with the explanation of the study procedures at session 1 on a scale of 4 (very satisfied—all components of the study were clearly explained) to 1 (very unsatisfied—all components of the study were poorly explained).

Participants were also asked if they experienced any major difficulties with their phones, if they experienced any feelings of discomfort or paranoia due to the study app running on their smartphones, or if there were any major changes in how they used their smartphones during the study period (yes or no). If they answered “yes” to changes in smartphone use, participants selected all applicable options, including “I used my phone less/more overall,” “I charged my phone less/more,” “I carried my phone with me less/more,” and “other.” Finally, troubleshooting contacts between study staff and participants were tracked and reported as part of feasibility findings.

#### Validators

##### Overview

Validation measures administered at session 1 included neuropsychological tests used widely in the clinical diagnosis of MCI and dementia; self- and informant-report measures of cognitive decline and everyday functioning; questionnaires pertaining to mood; and measures of gait speed, geospatial life space, and community participation. These measures have demonstrated strong psychometric properties and were therefore used together as validation comparisons against digital phenotyping data. More details are provided in the subsequent sections.

##### Neuropsychological Tests

The neuropsychological test battery included the Hopkins Reading Test [87] as an estimate of premorbid intellectual ability (IQ), the Mini-Mental State Examination [88] as a global cognitive screener, and tests of attention (Trail Making Test-Part A [89] and Wechsler Memory Scale—Revised Digit Span Forward [90]), processing speed (Salthouse Letter and Pattern Comparison [91]), executive function (Trail Making Test-Part B [89] and Wechsler Memory Scale—Revised Digit Span Backward [90]), episodic memory (Hopkins Verbal Learning Test—Revised Delayed Recall [92] and Brief Visuospatial Memory Test-Revised Delayed Recall [93]), and language (Animal Fluency [94] and Boston Naming Test 30-item version [95]). Raw scores of each test were transformed into demographically corrected *t* scores using the Calibrated Neuropsychological Normative System [94], adjusting for age, sex, education, and estimated premorbid IQ (ie, Hopkins Reading Test score), which enabled more accurate estimation of cognitive ability within our diverse sample. An average *t* score was computed for the 2 tests within each cognitive domain to generate composite scores for attention, processing speed, executive function, delayed memory recall, and language abilities to streamline presentation of results.

##### Reported Cognitive Decline and Everyday Functioning

Self-reported cognitive decline was collected using the Everyday Cognition Scale-Short Form [96]. Self- or informant-reported everyday functioning was captured with the Functional Activities Questionnaire (FAQ [97]). Self-reported FAQ was used for participants with healthy cognition, and informant-reported FAQ was used for participants with MCI or dementia. Higher scores on these measures indicate more cognitive decline and more functional impairment, respectively.

##### Mood

Mood symptoms were indexed using the 15-item Geriatric Depression Scale (GDS [98]), a widely used self-report measure of depressive symptoms among older adults that requires participants to indicate whether they experience a list of common depression symptoms in a “yes/no” format. Raw scores were transformed into demographically corrected *t* scores using the Calibrated Neuropsychological Normative System as mentioned earlier. Higher *t* scores indicate higher levels of depression.

##### Gait Speed, Life Space, and Community Participation

The Timed Up and Go Test (TUG) was administered at session 1 as an objective measure of gait speed. This task measures the time it takes to rise from a chair, walk 10 feet, turn, walk back to the chair, and sit down. It is widely used to examine balance, functional mobility, gait speed, and fall risk in older adults [99,100]. Participants also completed the University of Alabama at Birmingham Life-Space Assessment (LSA), a self-report measure of mobility for community-dwelling older adults [101]. It captures the level of independence and spatial extent of a person’s life over the preceding month and has shown strong associations with mobility within the home and community and with performance of activities of daily living [102]. Constricted life space has also been associated with risk for MCI and dementia [103,104]. The Australian Community Participation Questionnaire (ACPQ) 15-item version was administered as an additional measure of concurrent validity and assesses the extent to which someone engages in a range of community activities. Subscales include contact with immediate household, extended family, friends, and neighbors; participating in organized community activities; taking an active interest in current affairs; and religious observance [105]. An index of breadth of participation across the 7 domains was derived using a mean-split procedure for each domain, followed by summing these scores to generate an overall index ranging from 1 to 7 (as described by Brett et al [106]).

#### Other Participant Features

##### Demographic

Demographic data included participants’ self-reported biological sex assigned at birth, age, race, ethnicity, current living status (alone or with others), current occupational status, educational attainment, and other information related to socioeconomic status (eg, highest household annual income).

##### Technology Use

Participants completed a 6-item Habitual Smartphone Behavior subscale [107] to assess smartphone use patterns, providing responses ranging from “strongly agree” to “strongly disagree” to questions such as “Smartphone usage is part of my daily routines.” We also asked participants, “Do you usually have your phone with you when you leave home?” (smartphone portability), to which they could reply, “Yes- I almost never leave my house without my phone,” “In between—I leave my house without my phone about half the time,” or “No, I often leave my house without my phone.” The Mobile Device Proficiency Questionnaire was administered as a measure of digital literacy [108]. Participants also indicated their smartphone operating system (Android vs iOS).

##### Seasonal and COVID-19 Factors

Dates of study participation were collected and coded as winter, fall, spring, and summer to explore potential seasonal effects on mobility habits. Because study participation took place during the COVID-19 pandemic for some participants, we asked about the impact of the COVID-19 pandemic on social participation, routines, and mobility behaviors at the time of study participation. Participants were asked, “On a scale of 1-5, how isolated or cut off from family and friends are you feeling due to limited/canceled social gatherings resulting from COVID-19?” “On a scale of 1-5, how disruptive has the COVID-19 pandemic been to your daily routines and activities?” and “On a scale of very much limited to very much expanded, how much has the COVID-19 pandemic changed your mobility/your movements outside of the home?”

##### Self-Reported Health Changes During the Study Period

At the end of the study period during session 2, participants reported whether there were any changes in their overall health during the study period by responding to a single question on the debriefing survey. Response options included (1) yes, significant change; (2) yes, a little change; or (3) no change. If they endorsed any change in health, they were given the option to elaborate.

#### Digital Phenotyping (mindLAMP)

Though the mindLAMP app enables the collection of a wide array of deidentified passive and active data, this study focused on passively obtained GPS data. mindLAMP was configured to continuously record the device’s GPS coordinates at a maximum frequency of 1 Hz. Raw data outputs include latitude, longitude, altitude, and the coordinates’ estimated accuracy. At study completion, these raw data were extracted from the study server and processed into daily summary features (Table 1) using a publicly available R script developed by Barnett and Onnela [109,110]. GPS data from smartphone devices are prone to large amounts of missing data [111]; therefore, advanced multiple imputation methods based on weighted resampling of the observed data were used to account for missingness before feature calculation. These imputation methods are automatically incorporated within the aforementioned processing script and are described in detail in the study by Barnett and Onnela [109].

**Table 1:** Daily GPS features generated from mindLAMP raw data.

Category and feature		Feature description
**Activity**		
	Distance traveled	The sum of all flight lengths that day (m)
	Radius of gyration	Average distance (m) a person is from their center of mass (average position) on a given day
	Maximum diameter	Maximum diameter (m); longest pairwise distance between any 2 pause locations that occur that day
	Maximum distance home	Maximum distance from home (m); distance between home and farthest pause location from home that day
	Average flight length	The average length of all flights that day (m)
	Average flight duration	The average duration of all flights that day (s)
**Inactivity**		
	Home time	Time spent at home (min); amount of time spent that day within 200 m of home (the significant location with the largest total amount of time between 9 PM and 6 AM throughout the study period).
	Probability paused	Fraction of time a person is stationary (paused) during a day, relative to time spent mobile or in flight.
**Routine**		
	Circadian routine	Physical circadian routine; the fraction of time a person is in the same place (within a 200 m radius) at the same time of each day throughout the study period. Ranges from 0=completely different routine to 1=identical routine.
	Weekend circadian routine	Physical circadian routine weekend or weekday stratified; similar to circadian routine except comparisons are stratified by grouping together weekends and grouping together weekdays. Higher scores reflect greater overall routine.
**Location diversity**		
	Significant location entropy	Location entropy across a person’s significant locations for the day; large values indicate spreading time out across many different locations fairly evenly for that day; small values indicate a concentration at a few significant locations.
	Significant locations visited	The number of significant locations a person is within 200 m of that day. Determined using K-means on the set of all pause locations with a minimum duration of 10 minutes (longer pauses given additional weight and no 2 cluster centers within 400 m of one another).
**Other**		
	Minutes missing	The number of minutes of missing data preimputation in a person’s GPS mobility trace that day (min)

Supplementary materials from Barnett and Onnela [109] give a full description of GPS features and the methods used to calculate each. A flight is defined as a segment of linear movement; pauses are periods when a person does not move; and curved movement is approximated by multiple sequential flights.

### Statistical Analyses

#### Preliminary Processing

**Figure 2:**
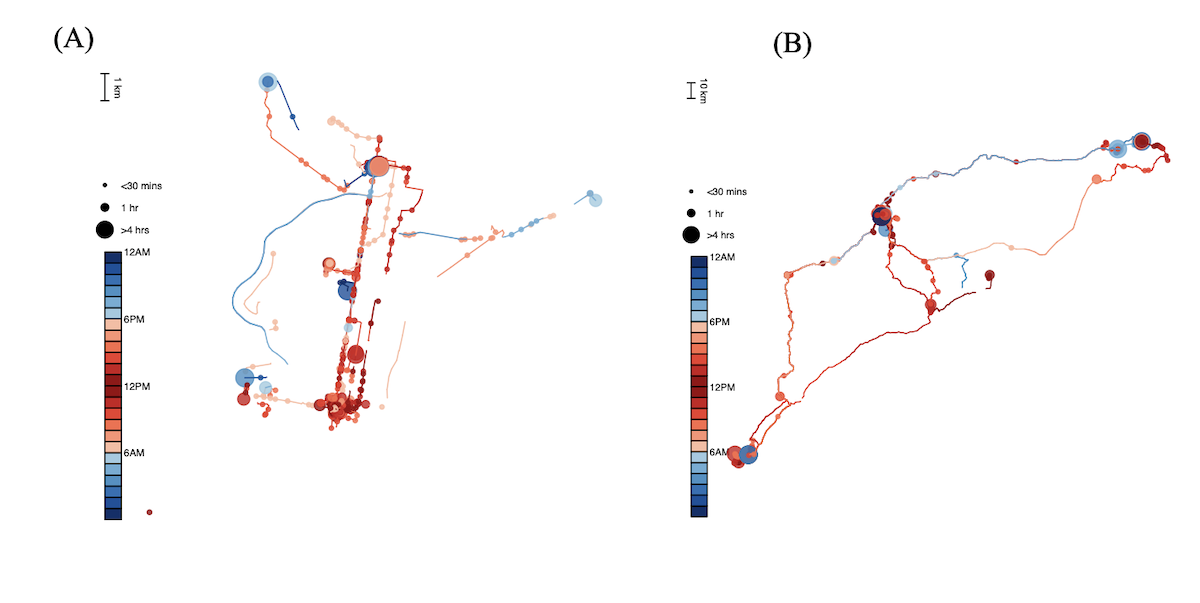
Example mobility plots depicting overall trajectories over 4 weeks for 2 participants. Solid lines depict flights from one location to another; circles indicate periods spent stationary, with larger sizes reflecting more time spent stationary at that location, color-coded by the time of day. (A) A 67-year-old woman living in northern Philadelphia. (B) A 70-year-old woman living in a suburb of Philadelphia.

The individual mobility traces derived from GPS data were visually inspected by examining each participant’s deidentified mobility plots averaged across the study period (Figure 2). These plots are generated automatically through our processing script and enable visualization of overall mobility habits, amount of time spent at various locations, and time of day. Following visual inspection, daily mobility features (Table 1) were collapsed into monthly overall average and monthly (day-to-day) individual SD (iSD) for each participant to generate estimates of mean mobility and mobility IIV across the study period. For example, 30 days of daily distance traveled estimates for 1 participant were reduced to an average distance traveled per day, and an iSD of daily distance traveled across the 30-day study period. This approach enabled the examination of a priori hypotheses as an important first step in the validation of GPS data. All variables (GPS and validation measures) with highly skewed distributions were transformed using log(x+c) transformation to reduce the influence of outliers and make parametric analyses more robust. Pearson correlation analyses were used to explore relations among individual GPS features.

Other preprocessing steps were completed in individual cases. Specifically, some participants had unanticipated travel during their scheduled study period (which was an exclusion criterion for validation purposes). In these cases, we extended their study duration and excluded days spent traveling from the raw data before feature extraction. In total, 3 (8%) of the 37 participants reported major unanticipated health changes during the study period (1 (3%) reported eye surgery, 1 (3%) reported a major fall with loss of consciousness, and 1 (3%) reported a fall with head trauma and COVID-19 infection) and were excluded from preliminary validity analyses in this study, because major health events during passive data collection would confound relations with baseline validators.

#### Primary Analyses

Descriptive statistics were first used to examine feasibility and acceptability outcomes across the entire sample (N=37; aim 1). Next, Pearson or Spearman correlation analyses were used to examine the relations between the mean (monthly average) and IIV (monthly iSD) of each mobility feature against validators collected at session 1 (ie, cognition, everyday function, mood, and mobility; aim 2). All validity analyses were conducted across the entire sample after excluding 3 participants with major health events (final n for aim 2 validation analyses=34). Given the small sample size, we interpreted correlation coefficients with *r*>0.3 as meaningful, regardless of statistical significance. Correlations surviving Bonferroni correction of *P*<.0038 (α corresponding to .05/13 GPS features) are noted to account for multiple comparisons. As mentioned earlier, we tested several a priori hypotheses based on our proposed conceptual framework (ie, the VIBE model, see Textbox 1). Specifically, we predicted that GPS mean-level activity metrics would show a positive linear relation with global cognition, function, and mood, whereas GPS IIV metrics would show a negative relation with cognition, function, and mood. Hypotheses from the original VIBE model were adjusted to consider trends along the continuum from healthy cognition to MCI only (excluding trends from MCI to dementia), given this study sample only included 1 participant with dementia. We also predicted a positive linear relation between GPS mean-level activity metrics and objective and subjective measures of gait speed, life space, and community participation that would reflect concurrent validity.

#### Exploratory Analyses

A series of exploratory analyses examined relations between GPS metrics and other participant factors such as demographics, technology habits, and smartphone type, as well as environmental factors (eg, COVID-19 and seasonal impact). Our goal was to better understand what intrinsic and extrinsic factors are associated with GPS trajectories in older adults so that future studies can consider these variables as potential covariates or moderators. This is important given the high heterogeneity in individual features and incidental factors that may impact the generalizability of between-group differences in GPS trajectories. Spearman correlation analyses were used for continuous, dichotomous, and ordinal variables, and 1-way ANOVA was used for categorical variables with a Welch test for unequal group variances.

To explore the utility of a GPS composite score, a Gaussian mixture modeling (GMM) approach was used to generate a nuanced yet singular representation of mobility trajectories. For each participant’s set of daily GPS features (Table 1), we fit a GMM with k=3 mixture distributions to allow for flexible modeling with consideration of differences in the distribution for each person due to various factors (eg, weekend and weekday differences). The choice of k=3 was determined to maximize clustering complexity subject to our sample size limitations to avoid overfitting. This method also allowed us to reduce the full set of GPS features to a single dimension. Next, we created a distance matrix between each pair of participants in the sample by calculating the integral of the squared distance between each GMM density (the larger the distance, the more different the pair’s overall mobility patterns). After calculating the distance matrix, we used multidimensional scaling (MDS) to extract a 1D representation of this distance matrix (hereafter termed “MDS1”), akin to a principal component [112]. This MDS1 metric is a relative measure that represents the similarity of overall mobility patterns across participants. For example, if 2 individuals have similar MDS1 values, their overall mobility patterns are similar, whereas 2 individuals with very different MDS1 values demonstrate different mobility patterns. The MDS1 variable was used in exploratory correlation analyses to identify whether patterns of mobility relate to validators, in contrast to total amounts of variability in individual mobility features.

### Ethical Considerations

All aspects of the study protocol received ethics approval from the institutional review board at Temple University (protocol number 27013). As described earlier, all participants provided informed consent, were given the option to opt-out of study participation at any time, and were compensated for their time and effort. Study data were deidentified according to privacy procedures outlined earlier. Participants were compensated US $50 upfront for their participation.

## Results

### Participant Characteristics

A total of 37 individuals participated in our study between April 2022 and January 2024. The full sample was included in the analysis of feasibility and acceptability outcomes, whereas a subset (n=34, 92%) was included in preliminary validity analyses after excluding those who experienced major unexpected health changes. The 3 (8%) participants excluded due to major health events were aged on average 70.9 (SD 6.9) years, 67% (2/3) were female, 100% (3/3) identified as non-Hispanic White, and they completed on average 16.9 (SD 1.8) years of education. In addition, 2 (67%) of the 3 participants were Android users, and all had healthy cognition.

Of the validation subset (n=34), participation was distributed fairly evenly across all 4 seasons: 6 (18%) in the winter, 10 (29%) in the fall, 9 (26%) in the spring, and 9 (26%) in the summer. Participants’ age ranged from 63 to 85 (mean 71.6, SD 5.5) years and they were on average highly educated (mean 16.4, SD 2.7 years; range 10-20 years). A majority of participants identified as female (23/34, 68%) and non-Hispanic or Latinx (33/34, 97%). A total of 56% (19/34) participants identified as White, 35% (12/34) as Black or African American, and 6% (2/34) as Asian. Most participants lived with others (23/34, 68%) and were retired (27/34, 79%). The majority (26/34, 76%) of participants were iPhone users, whereas 8 (24%) were Android users. Most participants met diagnostic criteria for healthy cognition (n=28, 82%) with a minority meeting criteria for MCI (n=5, 15%) or mild dementia (n=1, 3%). Scores on the Mini-Mental State Examination ranged from 24 to 30 (mean 28.3, SD 1.3), and according to the FAQ, participants on average experienced minimal difficulties with everyday functioning (mean 1.5, SD 2). Average responses on the GDS revealed low levels of self-reported depression (mean 1.5, SD 1.5) [113]. Participant demographic characteristics are detailed in Table 2, and scores on validation measures of cognition, everyday functioning, mood, and mobility are outlined in Table 3.

**Table 2:** Participant demographics. Includes data from subset (n=34) included in validation analyses.

Demographics		Values
Age (y), mean (SD)		71.6 (5.5)
Education (y), mean (SD)		16.4 (2.7)
**Sex, n (%)**		
	Male	11 (32)
	Female	23 (68)
**Race, n (%)**		
	Asian	2 (6)
	Black or African American	12 (35)
	White	19 (56)
	Not reported	1 (3)
English as a second language, n (%)		4 (12)
**Ethnicity, n (%)**		
	Hispanic or Latinx	1 (3)
	Not Hispanic or Latinx	33 (97)
**Living status, n (%)**		
	Live alone	11 (32)
	Live with others	23 (68)
**Current occupational status, n (%)**		
	Full-time employee, volunteer, or student	3 (9)
	Part-time employee, volunteer, or student	4 (12)
	Retired	27 (79)
**Highest annual household income (US $), n (%)**		
	<30,0000	2 (6)
	30,000-49,000	4 (12)
	50,000-69,000	3 (9)
	70,000-89,000	5 (15)
	90,000-99,000	3 (9)
	100,000-149,000	6 (18)
	≥150,000	8 (24)
	Prefer not to answer	3 (9)
**Phone type, n (%)**		
	iPhone	26 (76)
	Android	8 (24)
**Smartphone portability** ^a^ **, n (%)**		
	Yes	32 (94)
	Half the time	2 (6)
	No	0 (0)
**Consensus diagnosis, n (%)**		
	Healthy cognition	28 (82)
	MCI^b^	5 (15)
	Mild dementia	1 (3)

^a^Smartphone portability=usually has smartphone when leaves home (no=1, in between=2, and yes=3).

^b^MCI: mild cognitive impairment.

**Table 3:** Participant baseline validation measures. Includes data from subset (n=34) included in validation analyses.

Validation measures		Scores, mean (SD; range)
**Neuropsychological test (composite** ***t*** **scores)**		
	Global cognition (MMSE^a^)	52.4 (8.1; 37-67)
	Attention	52.1 (5.8; 38-66)
	Processing speed	54.5 (7.9; 36-74)
	Executive function	50.9 (7.3; 38-66)
	Memory	47.4 (11.1; 20-75)
	Language	50 (9.3; 28-72)
**Self- and informant-reported functioning**		
	Functional Activities Questionnaire	1.5 (2; 0-6)
	Everyday Cognition Scale-Short Form	1.3 (0.2; 1.0-1.8)
**Mood**		
	Geriatric Depression Scale (raw score)	1.5 (1.5; 0-5)
	Geriatric Depression Scale (*t* score)	52.2 (10.4; 38-76)
**Mobility**		
	Timed up and Go Test (s)	11 (4; 6-29)
	Life-Space Assessment	78.5 (20.1; 36-114)
	Australian Community Participation Questionnaire	3.8 (1.8; 1-7)

^a^MMSE: Mini-Mental State Examination.

### Feasibility and Acceptability (Primary Aim 1)

All 37 (100%) participants who began the study completed the 4-week monitoring period and session 2 without requesting to withdraw. Participants scored on average 97% (SD 5.7%) on the comprehension of consent quiz. In total, 2 (5%) participants had an initial score of 80% (8/10 questions correct), 7 (19%) scored 90%, and 28 (76%) scored 100% on their first attempt. The most frequently incorrect item was “Using the mindLAMP app will help improve my cognitive functioning,” to which 5 (13%) participants answered “yes.” Better performance on the comprehension of consent quiz was associated with higher education (*r*_s_=0.65; *P*<.001) and differed across racial groups (*F*_2,33_=8.4; η^2^=0.34; *P*=.001), with better performance among White participants versus Black participants according to post hoc comparisons (*P*=.02). When including education as a covariate, group differences for race remained statistically significant, though a lower effect size was noted (*F*_2,32_=3.3; η^2^=0.17; *P*=.049). Performance on the quiz was not associated with age, English as a second language, or cognitive status (all *P* values >.05).

Satisfaction ratings on the debriefing questionnaire at session 2 (ie, responses to the question “How satisfied are you with the study team’s explanation of this study? Did the study team accurately convey what it would be like to participate in this study during the consent process at your first study visit?”) revealed high levels of satisfaction. Specifically, 84% (31/37) of participants reported they were “very satisfied,” and 16% (6/37) reported they were “satisfied.”

Regarding new issues with or changes to phone use, 92% (34/37) of participants reported they did not experience any new problems using their smartphone during the study period. However, 1 (3%) participant experienced technical issues that were determined to be unrelated to the study application, 1 (3%) reported that some of their text messages were disrupted (unrelated to the study app as we did not collect information from text messages), and 1 (3%) reported that their phone was “a little slow and lack of charge.” In total, 97% (36/37) of participants reported they did not experience any feelings of being uncomfortable, suspicious, or paranoid due to the study app running on their smartphone. Although participants were instructed to go about their daily lives and smartphone use as they normally would, 46% (17/37) of participants reported there were major changes in how they used their smartphone during the study period. Specifically, 3% (n=1) used their phone less overall, 41% (n=15) charged their phone more, 5% (n=2) carried their phone less, and 14% (n=5) carried their phone more.

Troubleshooting contacts related to missing GPS data were infrequent. During the entire study period across 37 participants, only 6 incidents were logged affecting 5 (14%) unique participants, with causes including (1) Android phone went into “safe mode,” (2) location permissions reset from “always” to “only while using app,” (3) outdated version of mindLAMP installed on phone, (4) low battery mode enabled, and (5) mindLAMP app was accidentally deleted. All incidents were promptly resolved by the study coordinator remotely by speaking with the participant over the phone to guide them to either reconfigure their phone settings or re-download and login to mindLAMP.

### Validity Analyses (Primary Aim 2)

#### Preliminary Analyses

Examining the untransformed GPS data revealed that on average each day, participants spent 1074 (SD 192) minutes at home (ie, about 18 hours), traveled 42,676 (SD 34,694) m, spent time at 1.56 (SD 0.54) unique locations, and had 423 (SD 352) minutes of missing GPS data (approximately 29% of the day, which reflects relatively high data quality and frequency relative to other smartphone digital phenotyping studies using interval sampling approaches [111]). Correlations among GPS features revealed strong associations among features reflecting activity (distance traveled, radius of gyration, maximum diameter, and maximum distance from home), which were negatively associated with features reflecting inactivity (time spent at home and stationary time) and the 2 indices of physical circadian routine. Significant locations visited and significant location entropy were intercorrelated, suggesting a distinct construct related to location diversity. These associations together support the conceptual GPS feature categories outlined in Table 1 and are used throughout to streamline the presentation of results. Tables S1 and S2 in Multimedia Appendix 2 present descriptive statistics of GPS data and intercorrelations among all GPS features.

#### Relations Between Average Mobility Features and Measures of Cognition, Mood, and Everyday Function

We predicted significant relations between monthly average GPS activity features and baseline neuropsychological measures, mood, and everyday function, such that greater overall mobility would be associated with better performance on neuropsychological tests, less depression, and less reported cognitive and functional decline. Most correlations were in the predicted direction (Table 4; results for individual neuropsychological tests and function questionnaires are reported in Table S3 in Multimedia Appendix 2).

Correlations with neuropsychological composites and individual tests showed that greater GPS activity was associated with better scores on the language composite (Table 4) and Digit Span Forward test (Table S3 in Multimedia Appendix 2). GPS measures of inactivity and physical circadian routine were associated with lower language composite and individual test scores. Of note, correlations between greater physical circadian routine and lower language scores were the only relations to survive correction for multiple comparisons (*P*<.0038; Table 4; Table S3 in Multimedia Appendix 2). Associations between GPS activity features and the memory composite were not significant and weak but were in the opposite direction of most other neuropsychological composites. Analyses of individual neuropsychological tests showed greater average flight length was associated with worse delayed verbal recall (*r*=–0.38, *P*=.03; Table S3 in Multimedia Appendix 2), but the correlation coefficient was not statistically significant after correction for multiple comparisons.

Regarding mood, more inactivity (eg, more home time), greater routine, and less location diversity were associated with greater depression symptoms (Table 4). Less location diversity was also associated with greater reported functional impairment (FAQ; *r=*–0.36, *P*=.04; Table S3 in Multimedia Appendix 2). However, the associations between mobility features and self-reported mood and function did not survive correction for multiple comparisons. Correlations between GPS features and self-reported cognitive decline on the Everyday Cognition Scale-Short Form were weak and not statistically significant (Table S3 in Multimedia Appendix 2).

**Table 4:** Bivariate Pearson correlations between monthly average GPS features and neuropsychological measures (*t* scores)^a^.

Category and GPS monthly average feature		Global cognition (MMSE^b^)	Attention	Processing speed	Executive function	Memory	Language	GDS^c^
**Activity**								
	Distance traveled	0.15	0.33^d^	–0.01	0.07	–0.17	0.33^d^	–0.24
	Radius of gyration	0.21	0.25	–0.03	0.11	–0.10	0.34^d^	–0.21
	Maximum diameter	0.19	0.33^d^	0.01	0.10	–0.12	0.35^d,e^	–0.20
	Maximum distance home	0.22	0.27	0	0.08	–0.10	0.43^d,e^	–0.19
	Average flight length	0.22	0.26	–0.05	0.03	–0.27	0.38^d,e^	–0.33^d^
	Average flight duration	–0.05	0.15	–0.10	0.07	–0.01	0.16	0.01
**Inactivity**								
	Home time	–0.08	–0.11	0.22	0.16	0.17	–0.45^d,f^	0.38^d,e^
	Probability paused	–0.12	–0.28	0.13	–0.02	0.11	–0.22	0.29
**Routine**								
	Circadian routine	–0.07	–0.11	0.16	0.16	0.20	–*0.51*^d,f,g^	0.40^d,e^
	Weekend circadian routine	–0.08	–0.12	0.14	0.15	0.19	–*0.53*^d,f,g^	0.38^d,e^
**Location diversity**								
	Significant location entropy	0.04	0.20	–0.09	–0.06	–0.25	0.23	–0.36^d,e^
	Significant locations visited	–0.20	0.26	–0.02	–0.21	–0.14	0.07	–0.35^d,e^
**Other**								
	Minutes missing	0.28	0.47^d,f^	0.08	–0.01	–0.24	0.37^d,e^	–0.39^d,e^

^a^Data represent effect size as measured by bivariate Pearson correlation coefficients (*r*), whereby 0.10, 0.30, and 0.50 represent small, moderate, or large effects, respectively. All neuropsychological measures reflect *t* scores corrected for age, sex, education, and estimated premorbid IQ.

^b^MMSE: Mini-Mental State Examination.

^c^GDS: 15-item Geriatric Depression Scale *t* score.

^d^Moderate to large effect size.

^e^*P*<.05 (2-tailed).

^f^*P*<.01 (2-tailed).

^g^Italics indicate correlation coefficients surviving Bonferroni correction (*P*<.0038).

#### Relations Between Average GPS Features and Measures of Gait Speed, Life Space, and Community Participation

Contrary to our hypotheses, performance on the TUG measure of gait speed was not associated with any of the average GPS features (Table 5). By contrast, self-reported measures of geospatial life space (LSA), and community participation (ACPQ) were associated with many GPS features. Overall, greater GPS activity was associated with more self-reported life space and more community participation, whereas greater inactivity and physical circadian routine were associated with less geospatial life space and less community participation. Correlations between greater radius of gyration, less physical circadian routine, and greater geospatial life space survived correction for multiple comparisons (*P*<.0038; Table 5). Relations with the ACPQ were driven by the domains of extended family and friends (eg, participants who reported they tend to visit friends more often demonstrated significantly higher levels of GPS activity and lower physical circadian routine indices; *P*<.0038; Table S5 in Multimedia Appendix 2).

**Table 5:** Bivariate Spearman correlations between average GPS features and measures of gait speed, life space, and community participation^a^.

GPS monthly average feature				TUG^b^		LSA^c^		ACPQ^d^
**Activity**								
	**Distance traveled**							
		*r*	–0.16		0.40^e^		0.34^e^	
		*P* value	.37		.02		.048	
	**Radius of gyration**							
		*r*	–0.17		*0.52* ^e,f^		0.39^e^	
		*P* value	.35		.002		.02	
	**Maximum diameter**							
		*r*	–0.18		0.44^e^		0.40^e^	
		*P* value	.31		.009		.02	
	**Maximum distance home**							
		*r*	–0.11		0.44^e^		0.42^e^	
		*P* value	.54		.008		.01	
	**Average flight length**							
		*r*	–0.05		0.23		0.29	
		*P* value	.77		.18		.10	
	**Average flight duration**							
		*r*	0.01		0.34^e^		–0.09	
		*P* value	.94		.052		.62	
**Inactivity**								
	**Home time**							
		*r*	0.12		–0.42^e^		–0.39^e^	
		*P* value	.49		.01		.02	
	**Probability paused**							
		*r*	0.13		–0.48^e^		–0.24	
		*P* value	.46		.004		.18	
**Routine**								
	**Circadian routine**							
		*r*	0.12		–0.45^e^		–0.45^e^	
		*P* value	.50		.007		.008	
	**Weekend circadian routine**							
		*r*	0.18		–*0.50*^*e,*^^f^		–0.48^e^	
		*P* value	.32		.002		.004	
**Location diversity**								
	**Significant location entropy**							
		*r*	–0.15		0.34^e^		0.30^e^	
		*P* value	.40		.052		.08	
	**Significant locations visited**							
		*r*	–0.18		0.29		0.21	
		*P* value	.31		.09		.23	
**Other**								
	**Minutes missing**							
		*r*	0.17		0.16		0.32^e^	
		*P* value	.34		.35		.07	

^a^*r* represents effect size as measured by bivariate Spearman correlation coefficient, whereby 0.10, 0.30, and 0.50 represent small, moderate, or large effects, respectively.

^b^TUG: Timed Up and Go Test.

^c^LSA: Life-Space Assessment.

^d^ACPQ: Australian Community Participation Questionnaire.

^e^Moderate to large effect size.

^f^Italics indicate correlation coefficients surviving Bonferroni correction (*P*<.0038).

#### Relations Between GPS Variability and Measures of Cognition, Mood, and Everyday Function

We predicted a negative relation between day-to-day variability in GPS features and baseline measures of cognition, mood, and everyday function, such that greater IIV would be associated with lower neuropsychological test scores, more depression, and more reported cognitive and functional decline. Contrary to our hypotheses, we saw that higher variability in most GPS features (ie, greater day-to-day iSD in mobility habits) was associated with *better* scores on neuropsychological measures of attention and language, as seen in Table 6 and in Tables S6 and S7 in Multimedia Appendix 2. Correlations between greater IIV in home time and physical circadian routine and higher language composite scores survived correction for multiple comparisons (*P*<.0038; Table 6). Again, relations with memory were in the opposite direction, such that higher GPS IIV in location entropy was associated with a worse memory composite. Analyses with individual tests showed significant associations between higher GPS IIV in average flight length and worse verbal memory and higher GPS IIV in location entropy and worse visual memory (*P*<.0038; Table S7 in Multimedia Appendix 2). Regarding relations with mood, greater IIV in location diversity was associated with less depression, though results did not survive correction for multiple comparisons. This was contrary to expectations but consistent with the aforementioned results suggesting greater variability in mobility habits is overall beneficial. GPS IIV was not significantly associated with self-reported cognitive decline or functional impairment, though results are directionally consistent, such that greater IIV was weakly related to less reported functional decline (Table S7 in Multimedia Appendix 2).

**Table 6:** Bivariate Pearson correlations between monthly IIV GPS features and neuropsychological measures (*t* scores)^a^.

Category and GPS monthly IIV^b^ feature		Global cognition (MMSE^c^)	Attention	Processing speed	Executive function	Memory	Language	GDS^d^
**Activity**								
	Distance traveled	0.22	0.24	0.01	0.15	–0.08	0.37^e,f^	–0.14
	Radius of gyration	0.27	0.15	0	0.12	0.05	0.33^e^	–0.07
	Maximum diameter	0.27	0.20	0.06	0.13	0.03	0.37^e,f^	–0.06
	Maximum distance home	0.27	0.16	0.03	0.12	0.04	0.40^e,f^	–0.06
	Average flight length	0.27	0.16	0.04	0.18	–0.32^e^	0.35^e,f^	–0.28
	Average flight duration	0	0.06	–0.08	0.10	–0.03	0.18	0.02
**Inactivity**								
	Home time	0.23	0.15	–0.13	0	–0.18	*0.64* ^e,g,h^	–0.31^e^
	Probability paused	0.17	0.27	–0.10	0.13	–0.09	0.29	–0.10
**Routine**								
	Circadian routine	0.15	0.17	0.10	0.07	–0.02	*0.59* ^e,g,h^	–0.20
	Weekend circadian routine	0.16	0.13	–0.03	0.04	–0.02	*0.55* ^e,g,h^	–0.28
**Location diversity**								
	Significant location entropy	0.01	0.25	0.06	–0.09	–0.45^e,g^	0.35^e,f^	–0.47^e,g^
	Significant locations visited	–0.21	0.11	–0.07	–0.33^e^	–0.13	0.32^e^	–0.36^e,f^
**Other**								
	Minutes missing	0.29	0.06	–0.13	0.03	0	0.41^e,f^	–0.22

^a^Data represent effect size as measured by bivariate Pearson correlation coefficients (*r*), whereby 0.10, 0.30, and 0.50 represent small, moderate, or large effects, respectively. All neuropsychological measures reflect *t* scores corrected for age, sex, education, and estimated premorbid IQ. Table S6 in Multimedia Appendix 2 gives exact *P* values.

^b^IIV: intraindividual variability.

^c^MMSE: Mini-Mental State Examination.

^d^GDS: 15-item Geriatric Depression Scale *t* score.

^e^Moderate to large effect size.

^f^*P*<.05 (2-tailed).

^g^*P*<.01 (2-tailed).

^h^Italics indicate correlation coefficients surviving Bonferroni correction (*P*<.0038).

### Exploratory Analyses

#### Relations Between GPS Metrics and Participant Intrinsic and Extrinsic Factors

To inform future studies in the selection of covariates or moderating variables, we conducted exploratory correlations between GPS average and IIV features and various participant, environmental, and contextual factors (Tables S8 and S9 in Multimedia Appendix 2). Average GPS features were unrelated to several sociodemographic factors, including age, sex, and cohabitation status (all *P* values >.05). Higher education was associated with less location diversity only (*r*=–0.35; *P*=.04) but was not significant after correction for multiple comparison. By contrast, lifetime annual income and native language status appeared to be more relevant. Overall, higher lifetime income and native English language status were associated with greater levels of GPS activity, less routine, and greater location diversity (0.36≤|*r*|≤0.55). The association between higher lifetime income and greater GPS activity measures (radius of gyration and maximum distance from home) remained significant after correction for multiple comparisons (*P*<.0038; Table S8 in Multimedia Appendix 2).

Technology factors were largely unrelated to average GPS features. Phone type (iPhone vs Android) was unrelated to all features except for the overall number of minutes missing; iPhone users had significantly less missing GPS data than Android users, and the relation remained significant after correction for multiple comparisons (*r*=–0.64; *P*<.0038). More habitual smartphone use was associated with less location diversity only (*r*=–0.40; *P*=.02), though this relation did not survive correction for multiple comparisons. Whether or not participants typically carry their phone when leaving home (smartphone portability) was unrelated to all average GPS features, as was digital literacy as measured by the Mobile Device Proficiency Questionnaire (all *P* values >.05). Regarding the impact of COVID-19, individuals who reported more COVID-19–related isolation (ie, due to limited or canceled social gatherings) demonstrated significantly less location diversity (*r*=–0.51; *P*<.0038), whereas those who reported a greater expansion in mobility habits due to COVID-19 demonstrated more location diversity (*r*=0.34; *P*=.047), though this latter relation was not significant after correction for multiple comparisons.

One-way ANOVA of average GPS features was used to explore group differences across categorical demographic variables (race, occupational status, and season), with the Welch test for unequal group variances. Group differences for race (Black, Asian, and White) were observed only on the minutes missing feature (*F*_2,30_=6.32; η^2^=0.30; *P*=.005), with Black participants having greater amounts of missing GPS data than White and Asian participants according to post hoc comparisons (*P=*.003 and *P*=.02, respectively). A follow-up chi-square test was performed to examine the relationship between race and phone type given prior findings that Android users have greater missing data than iPhones [114]. The relation was significant, such that a greater proportion of Black participants owned Androids, *χ*^2^_2_=6.9, *P*=.03 (N=33), suggesting that group differences in missing data could be related to phone type.

**Figure 3:**
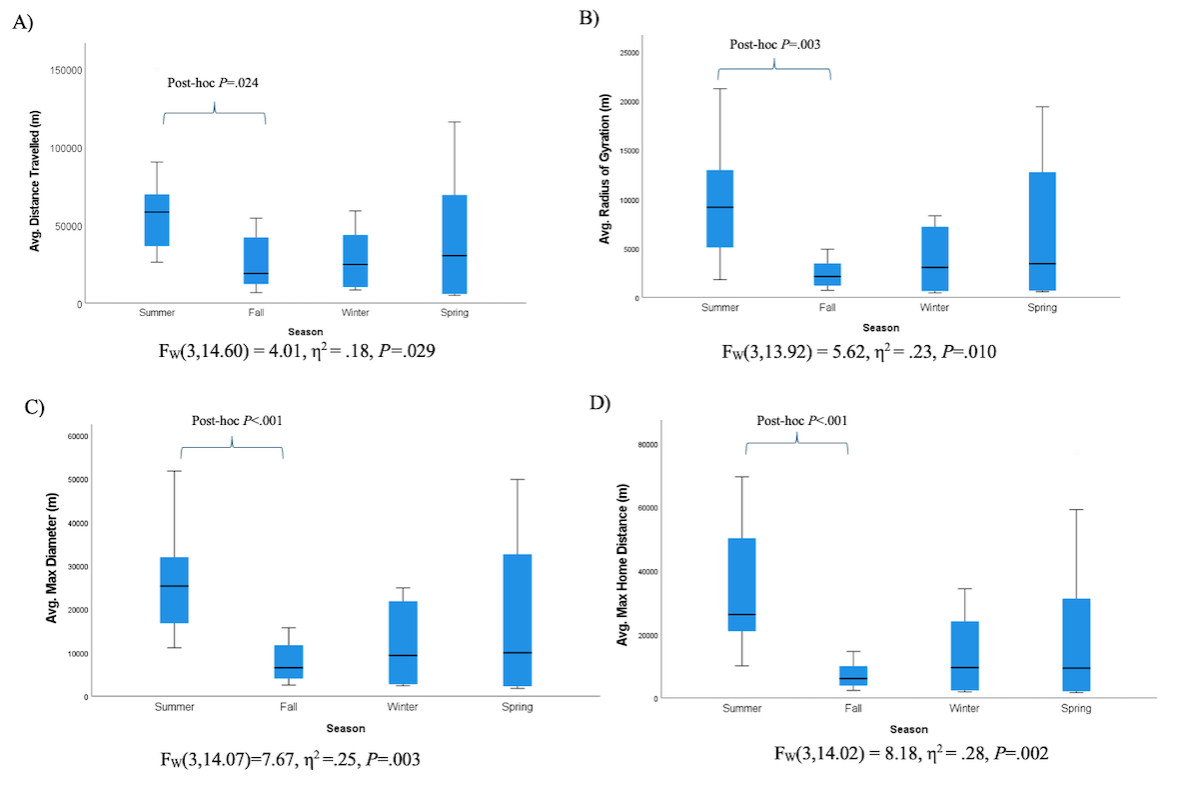
One-way ANOVA of GPS activity features by season.

No group differences in average GPS features were observed according to current occupational status (eg, retired and working full vs part-time). By contrast, several activity features were significantly different across the winter, fall, spring, and summer seasons (4.01≤*F*_3,30_≤8.8; 0.18≤η^2^≤0.28; all *P* values <.05). According to post hoc comparisons, these differences were driven by significantly greater GPS activity in the summer versus fall months (Figure 3).

Spearman correlations between GPS IIV features and the participant factors assessed earlier are detailed in Table S9 in Multimedia Appendix 2. Similar to the results with average GPS metrics, relevant sociodemographic factors appeared to be income and native language status. Higher lifetime income and native English language status were associated with more variability in activity, routine, and location diversity features (0.35≤|*r*|≤0.56), with associations between higher income and greater IIV in activity (radius of gyration, maximum diameter, and maximum distance from home) surviving correction for multiple comparisons (*P*<.0038).

Phone type was more prominently related to GPS IIV measures than GPS average measures; iPhone users showed greater GPS IIV than Android users on several GPS features (0.34≤*r*≤0.52), though only greater GPS IIV in minutes missing survived correction for multiple comparisons (*P*<.0038). Of note, iPhone ownership was also positively correlated with income (*r*=0.49; *P*=.005), making it difficult to determine whether higher levels of IIV among iPhone users relate to differences in phone type or to behavioral differences in lifestyle afforded by higher income. Regarding other technology factors, habitual smartphone use and smartphone portability were unrelated to GPS IIV metrics, whereas digital literacy was related to more variability in routine only (*r*=0.34; *P*=.047)—though this relation did not survive correction for multiple comparisons. Individuals who reported more COVID-19–related social isolation had significantly lower IIV in location diversity (ie, more day-to-day consistency in the number of locations visited; *r*=–0.55; *P*<.0038), whereas those who reported a greater expansion in mobility habits due to COVID-19 had greater IIV in location diversity (*r*=0.37; *P=*.03), though this latter relation was not significant after correction for multiple comparisons.

**Figure 4:**
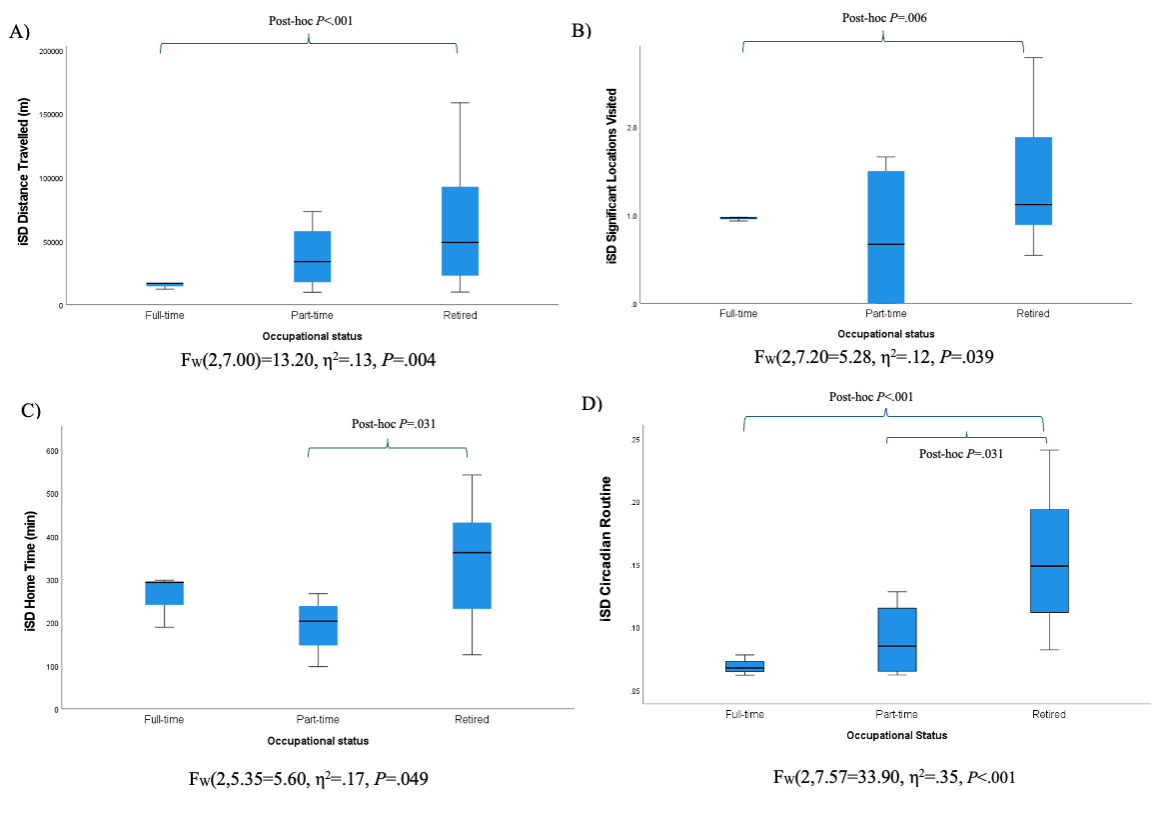
One-way ANOVA of GPS intraindividual variability features by occupational status.

One-way ANOVA of GPS IIV features across categorical demographic variables revealed a significant difference across racial groups on only 1 measure—IIV for flight length (*F*_W2,18.63_=11.96; η^2^=0.12; *P*<.001). This group difference was driven by lower IIV in Asian participants compared with both Black participants and White participants according to post hoc tests. Effects of current occupational status were observed on several IIV metrics (5.28≤*F*_2,31_≤33.90; 0.12≤η^2^≤0.35; all *P* values <.05), such that retired participants demonstrated significantly greater IIV in mobility habits compared to those working full- or part-time (Figure 4). Consistent with the results of GPS average metrics, several GPS IIV metrics differed according to the season of study participation, with significantly more IIV in activity features observed during summer months versus the fall and spring months (2.41≤*F*_3,30_≤3.78; 0.19≤η^2^≤0.27; all *P* values <.05).

#### Relative Mobility Patterns

A GMM, MDS approach was used to derive a relative mobility feature (MDS1), including all GPS features simultaneously. This allowed us to explore how similarity in mobility patterns, as reflected through a 1D value, related to validators of cognition, function, and mood. Relative mobility patterns were moderately associated with a cognitive measure of language (|*r|*=0.36; *P*=.04) and a global cognitive screener (|*r|*=0.31; *P=*.08; Table S10 in Multimedia Appendix 2). Relative mobility patterns were also associated with depression (|*r|*=0.39; *P=*.02) and community participation (|*r|*=0.51; *P*=.002). Of note, only the relation between MDS1 and community participation (ACPQ) survived correction for multiple comparisons (*P*<.0029). While the MDS1 feature represents a linear contrast combining multiple individual GPS features that excel at differentiating mobility patterns across the sample, this comes at the cost of interpretability of the associations related to MDS1 (eg, positive vs negative correlation coefficients are not meaningful); however, these results provide additional converging evidence that individuals with similar mobility profiles may have similar levels of underlying cognitive ability, depression, and community participation.

## Discussion

### Principal Findings

This study investigated a 4-week smartphone digital phenotyping protocol as a novel method to assess everyday cognition, function, and mood in a cohort of 37 older adults. Our preliminary, proof-of-concept results suggest that theoretically informed digital phenotypes of mobility are feasibly captured from older adults’ personal smartphones and are associated with clinically relevant data pertinent to cognitive aging and AD/ADRD. Findings and implications provide key insights to inform the design and interpretation of future studies using this method in larger, more diverse cohorts.

One of our primary aims was to examine the feasibility and acceptability of smartphone digital phenotyping among older adults. All participants completed the 4-week monitoring period without dropping out, and 97% (36/37) reported no feelings of discomfort during debriefing procedures. By contrast, almost half (17/37, 46%) reported changes in how they used their smartphone, with 41% (15/37) endorsing charging their phone more frequently. Battery drain was a communicated risk and is common in high-frequency continuous data collection, including GPS [111]. Increased phone charging behavior may limit the naturalistic aspect of this approach and will be important to address in future designs—particularly if battery power is considered as a digital biomarker in and of itself. Future studies should explore whether lower sampling frequencies are sufficient, as this can be adjusted via the LAMP platform and would lead to less battery drain. It is also likely that advances in smartphone battery life span will ultimately circumvent this issue; in the interim, participants may be provided with portable batteries for daily outings.

We were also interested in how participants would respond to and comprehend complex technical details of this study. Participants demonstrated good comprehension of study procedures, as demonstrated by an average score of 97% on the comprehension of consent quiz, and all reported satisfaction with the study team’s initial review of study procedures. These findings are encouraging given the dearth of studies investigating older adults’ attitudes and concerns about passive sensing technologies [78,115] and suggest that our upfront efforts to enhance privacy, security, transparency, and comprehension were effective [79]. However, despite high overall comprehension, we observed that higher education and identifying as White versus Black were associated with better performance on the comprehension of consent quiz—suggesting the language used in our materials may not be culture-free and should be revised using co-design or focus group approaches with increasingly diverse perspectives. Importantly, the most frequently incorrect item on the quiz pertained to potential benefits of study participation (“Using the mindLAMP app will help improve my cognitive functioning,” yes or no). Clear communication of potential benefits in research is a core ethical requirement, and future study materials should clarify expectations for potential benefits to facilitate trust, particularly among historically marginalized groups [116-119].

Regarding our second primary aim of establishing preliminary validity, we observed converging support that unobtrusively obtained movement trajectories from smartphones are related to established clinically relevant variables, including cognition, function, and mood. This is not surprising but is highly encouraging. Movement trajectories reflect many facets of everyday cognition, including the ability and motivation to travel outside the home, the degree of someone’s spatial routine, number of unique locations someone can visit, and the total amount and duration of movement—behaviors that require abundant cognitive and psychological resources. Here, individuals with better cognition, less functional impairment, and less depression did, in fact, demonstrate greater overall mobility according to several GPS features. Specifically, they traveled farther, spent less time at home, and had greater diversity in the locations they visited. Although these associations did not remain statistically significant after correction for multiple comparisons, these moderate-level effects in a relatively small sample are encouraging as they are consistent with our hypotheses and with prior studies showing greater physical activity, less time at home, greater life space (the extent of movement through the environment during daily functioning), and engagement in a variety of activities (environmental complexity) are associated with better cognition, less depression, and reduced risk for MCI and dementia or AD/ADRD [66,103,104,120-128].

Inconsistent with our hypotheses, individuals with better cognition demonstrated significantly *greater* day-to-day variability in GPS features and *less* physical circadian routine, and these associations did survive correction for multiple comparisons. We also saw that greater mobility variability and less routine were associated with less depression, although associations did not survive correction for multiple comparisons. Regarding depression, it is conceptually reasonable that more varied mobility habits could be protective against depression, and this has been demonstrated in at least 1 study of younger adults where lower location diversity was associated with more depression symptoms [129]. With respect to the significant negative associations between cognition and routine, it is possible that the older adults in our cohort with more cognitive difficulties intentionally engaged in more predictable and less demanding daily activities to compensate for underlying mild difficulties, leading to more consistent physical circadian routines and lower day-to-day variability in mobility habits. This is a pattern identified in the literature and is often recommended as an intervention in clinical practice [130-134].

The VIBE model predictions regarding variability were informed by observations that individuals with cognitive impairment demonstrate increased IIV compared with healthy controls while performing single, standardized tasks in the clinic or laboratory where task demands are the same for everyone. Greater IIV on constrained tasks reflects an inability to maintain consistent levels of performance [7,135,136]. This study instead involved unconstrained mobility habits, which individuals may modify to compensate for cognitive difficulties. Therefore, the observation of GPS IIV as indicative of positive rather than negative outcomes may be specific to unconstrained geospatial routines or to our relatively functionally healthy sample. We may still observe that greater IIV associates with worse outcomes when considering more fine-grained digital biomarkers (eg, diurnal phone use patterns and accelerometer-based sleep estimates) that are relatively more constrained, as has been identified in other studies examining IIV in gait speed, medication-taking routine, and computer use [137-139]. We may also see that greater IIV in broad everyday behaviors is a marker of resilience early on but becomes maladaptive in later stages of neurocognitive decline. Larger samples with greater heterogeneity in cognitive ability are needed to answer this question, in addition to longitudinal designs that would enable monitoring of within-person IIV trends over time. In the meantime, our relatively cognitively healthy sample provides insights into how variability behaves in early stages and unconstrained settings. Consistent with past reports of significant task and timescale effects on IIV [140], IIV in continuous mobility trajectories may be mechanistically distinct from IIV in constrained settings.

Concurrent validity was supported by strong and significant associations between mean-level GPS features and baseline scores on the LSA and the ACPQ—self-report measures of geospatial life space and community activity participation, respectively. These 2 constructs are highly relevant in the context of aging and AD/ADRD and represent key measures of risk. The LSA measures the extent, frequency, and independence of movement within and outside the home. Constricted life space has been associated with increased risk of AD, MCI, and cognitive decline in racially diverse groups and may represent an early functional marker of prodromal decline as individuals compensate for early subtle difficulties and limit their range of movement or activity complexity [103,104,130]. Social engagement—particularly leaving the home to visit friends—protects against social isolation, promotes cognitive reserve, and represents a complex activity requiring cognitive flexibility [141-145]. Thus, the ability to unobtrusively and longitudinally measure these key risk and resilience factors without the burden or bias of self- or informant-report represents a noteworthy application for smartphone-derived mobility trajectories. Minimal associations were identified between GPS features and the TUG measure of gait speed. It is possible that low heterogeneity in TUG scores or small variations in the administration of the TUG played a role. It is also likely that gait speed and coordination are lower-level features of mobility that are independent of broader mobility habits, at least within this sample of functionally independent older adults.

We examined many individual and contextual influences on mobility features to inform future studies in selecting covariates or moderating factors. This is a critical open question in the field of digital phenotyping [66], and our preliminary results provide important insights into which sociodemographic, contextual, and technological factors should be considered when interpreting these data. Age, education, sex, race, and cohabitation status appeared to be minimally associated with most mean-level and variability metrics, providing partial support for the objectivity of digital phenotyping features. Nonetheless, other sociodemographic factors, including higher lifetime income and native English language status, were moderately associated with several mobility metrics that appear to be advantageous (ie, greater activity and greater IIV). This could reflect an association between social determinants of health and access to transportation, opportunities for socialization outside of the home, or an ability to engage in spontaneous activities—and may suggest mechanisms for income and acculturation as resilience factors.

Other extrinsic factors with relatively clear and interpretable effects included season, COVID-19–related effects, and occupational status. Participants demonstrated greater activity, less home time, and more mobility variability in the summer months, which is reasonable given the increased leisure activities that typically occur in the summer. Therefore, seasonal differences are relevant if interpreting data in pre-post designs, suggesting investigators should aim to control for season or re-evaluate during the same season if possible. Participants reporting more COVID-19–related isolation visited fewer locations, whereas those reporting more COVID-19–related mobility expansion visited more. Individuals who were retired demonstrated more variable mobility habits than those working full or part time, which aligns with differential degrees of consistency depending on occupational status. In addition to shedding light on which factors are relevant when interpreting digital phenotyping data, these associations provide additional validation for the mobility features in this study.

Phone type was unrelated to all mean-level GPS metrics except for the number of minutes of missing data, which was significantly lower for iPhones. This finding is consistent with a previous study by Kiang et al [114] that identified lower rates of missing GPS data among iOS users and overall bodes well for the generalizability of mean-level metrics across different phone types and operating systems. Missing data were also higher among Black participants, which could be due to a higher proportion of Android ownership among Black participants in our study. Thus, controlling for phone type may be important when interpreting the minutes of missing data feature. iPhone users also demonstrated more variability in most GPS features, although only the missing data feature remained statistically significant after correction for multiple comparisons. Given iPhone ownership was positively associated with income, it is difficult to interpret whether increased variability among iPhone users was related to operating system factors, to aspects of resilience associated with income, or whether this reflects a spurious finding. Future studies with larger sample sizes should work to clarify these questions.

In considering the neuropsychological correlates of our GPS features, relations with cognition were strongest and most consistent for measures of language. In fact, relations between GPS features and the language composite were the only ones to survive correction for multiple comparisons (ie, less overall physical circadian routine, greater IIV in circadian routine, and greater IIV in home time were all strongly [|*r|*>0.5] associated with the language composite at *P*<.0038). This was somewhat unexpected given language abilities are typically not as critical to the completion of everyday activities compared to domains such as executive functioning [146,147]. Nonetheless, intact language functioning (specifically semantic access and retrieval) relies on left temporal and prefrontal integrity and connectivity, which are highly relevant neuroanatomical regions in the pathogenesis of AD. Interestingly, relations between GPS features and measures of memory were in the opposite direction compared to other cognitive domains; for example, more day-to-day variability in average flight length was associated with worse delayed memory performance. This finding may reflect inefficient planning and daily errors resulting in occasional backtracking when visiting common locations due to forgotten items. In general, differential relations between GPS features and measures of memory and language—2 cognitive domains implicated in AD pathology—suggest relations between GPS features and cognitive abilities may not be as straightforward as our model predicted. Future studies should continue to investigate whether mobility phenotypes are uniquely related to specific neuropsychological and neuroanatomical correlates, rather than focus on a global cognitive composite. This may involve developing even more fine-grained GPS features to capture distinct functional difficulties in everyday life (eg, backtracking and repetitive motions).

### Limitations and Strengths

Our study has several limitations that are worth noting. Our relatively small sample size of 37 individuals coupled with a relatively large number of analyses limits generalizability; thus, findings should be considered preliminary and proof-of-concept. Digital phenotyping is a new research area with relatively few established standards, yet preliminary validation of digital phenotypes has been reported in samples of under 50 participants [61,73,148,149] for ≤30 days of data collection [73,150-152]. Another major limitation is the restricted diversity of our cohort in terms of cognition and diagnostic severity (which precluded investigation of diagnostic group differences), demographics (ethnicity, education, and socioeconomic status), and severity of depressive symptoms. Furthermore, all participants lived within driving distance of Temple University and therefore reflected an urban and suburban cohort. In addition, individual data-processing exclusions were required to account for unanticipated travel and health events. Given our aim of establishing preliminary validity, there was a need to ensure participants met strict inclusion criteria, which included relative stability in their health and physical location. This level of control and oversight may be unrealistic in larger, longitudinal studies and may need to be replaced by advanced statistical methods in the future [153]. Finally, it is worth mentioning that mobility traces from smartphone GPS sensors are a proxy for actual movement trajectories and depend upon the participant having their smartphone on their body, requiring multiple imputation and inference for missing GPS data before feature calculation during preprocessing [109]. It is also critical that participants are the unique users of their smartphones, as others have mentioned issues with shared devices [154,155]. Sensors worn on the body may provide more accurate and reliable measures of mobility patterns but present other drawbacks (eg, may be perceived as more intrusive by participants, may disrupt existing habits, and are typically costly).

This study has several strengths. Our cohort was well characterized compared to many prior digital phenotyping studies, including a comprehensive neuropsychological battery with 10 individual neurocognitive tests, objective and subjective validation measures, and application of combined actuarial and consensus diagnosis criteria to accurately classify participants. Our study design and hypotheses were theoretically informed by a conceptual model, which improves the interpretability and replicability of our findings. Toward this aim, we examined a range of interpretable mobility features (ie, 13 monthly average features and 13 monthly iSD features) with correction for multiple comparisons before reducing features into a principal component (MDS1). Results from the singular MDS1 component provided additional evidence that individuals with similar cognitive, functional, and mood profiles may demonstrate similar mobility profiles. Although many GPS features were intercorrelated, the presence of differential and unique correlations suggests individual features may be useful in clarifying specific behaviors and should be preserved as we continue to learn more about what these features signal. In addition, relative mobility profiles similar to the singular MDS1 component may be useful in identifying and classifying individuals with similar levels of underlying community participation and functional resilience. With regard to our technical protocol, the use of an open-source platform and a publicly available data processing script facilitates replicability, which is key to ongoing validation efforts [156]. Attempts at minimizing missing data using an automated checking script represent another strength and should be incorporated in future studies given the impact of missing data on subsequent findings [66]. Finally, our device-agnostic approach leveraging personally owned smartphones versus a study-issued device represents a notable strength as it promotes the naturalistic, unobtrusive nature of this method and affords increased scalability.

### Future Directions

Future studies with larger and more diverse cohorts will be critical to replicate the present findings and address open questions. Given the lack of accessible and unbiased diagnostics available to individuals from low-income and marginalized groups [53-55,157], increased diversity in terms of race, ethnicity, educational attainment, and socioeconomic status is a priority for subsequent studies. Validating this method in more clinically heterogeneous samples will be critical to evaluate its ability to distinguish between age-related cognitive decline, neurodegenerative decline, and other medical or psychiatric conditions, and between various clinical and biological subtypes of dementia and ADRD. Both activity-level and IIV metrics should be evaluated, as variability appears to be fundamentally distinct from mean-level metrics and relates to AD risk [158]. Next steps will also investigate relations between validation measures and other sensors (accelerometer, device state, and steps) to extend the present findings and test our theoretical framework in behavioral features other than mobility trajectories. Machine learning or cluster approaches integrating multiple sensor streams may be helpful in determining clinically useful digital phenotypes, thereby reducing the analytic burden and narrowing the focus on clinically relevant, nonredundant features. Additional open questions include the test-retest reliability of digital phenotypes; the incremental utility of ecological momentary assessment, which can provide context to passive data [7,49,159]; determining the minimum necessary sensor sampling rate and duration to reduce battery drain; and evaluating within-day variability, diurnal patterns, and time of day effects on GPS features [26,49,75,138,160].

Because there is no ground truth for the application and interpretation of digital phenotyping data in aging and ADRD populations, additional studies are needed so that results can be compared across samples and insights can be consolidated to inform gold-standard approaches and normative data. As the field continues to evolve, it is likely that longitudinal monitoring of within-person fluctuations will be the ideal approach to capture individually relevant changes rather than population- or group-based approaches [7,63,156,161]. This is particularly relevant in the context of first-line primary care screening and ADRD clinical trials, where scalable risk detection, trial screening, and treatment response measures are direly needed. However, with longitudinal designs and increased sample sizes, it will be important to consider whether the data storage, processing, and interpretation burdens justify breaking from the status quo. Workflows that enhance the efficiency and scalability of this method will be critical, not just for research participants and patients but also for those collecting and interpreting the data [7,80,111].

### Conclusions

As the population of older adults continues to rise, efforts to identify new tools to detect risk for future cognitive decline and measure treatment response are critical, particularly as new pharmacological interventions gain approval. Current gold standard methods are costly, burdensome, not widely accessible, and not part of routine clinical care. Measures that are sensitive to cognitive decline that can be passively and affordably integrated into everyday life without burden, disruption, or self-report bias are needed to serve as a first-line approach. Our study demonstrates that unobtrusively obtained GPS movement trajectories from personal smartphones may in the future be one such first-line approach, enabling clinicians and researchers to efficiently assess cognitive status, mood, and dementia risk on a broader scale. Individuals with at-risk data profiles may ultimately be referred for more comprehensive evaluation and directed toward appropriate intervention or research settings, leading to cost savings, reduced burden, and faster access to care. Much work remains to be done before we determine how smartphone digital phenotyping can be integrated into our current health care system; however, preliminary results suggest that it is a worthwhile endeavor and serve to inform follow-up studies that are necessary to answer important, outstanding questions.

## Appendix

Multimedia Appendix 1 Comprehension of consent quiz.

Multimedia Appendix 2 Supplementary data and analyses.

## Abbreviations

ACPQ: Australian Community Participation Questionnaire
AD/ADRD: Alzheimer disease and related dementias
AD: Alzheimer disease
FAQ: Functional Activities Questionnaire
GMM: Gaussian mixture modeling
HIPAA: Health Insurance Portability and Accountability Act
IIV: intraindividual variability
iSD: individual SD
LAMP: Learn, Assess, Manage, and Prevent
LSA: Life-Space Assessment
MCI: Mild Cognitive Impairment
MDS: multidimensional scaling
REDCap: Research Electronic Data Capture
TUG: Timed Up and Go Test
VIBE: Variability in Everyday Behavior
