# Supplementary figures and images for: Mobility-Based Smartphone Digital Phenotypes for Unobtrusively Capturing Everyday Cognition, Mood, and Community Life-Space in Older Adults: Feasibility, Acceptability, and Preliminary Validity Study
Source: JMIR Hum Factors. 2024 Nov 22;11:e59974. doi: 10.2196/59974 (PMC11624463; doi:10.2196/59974)

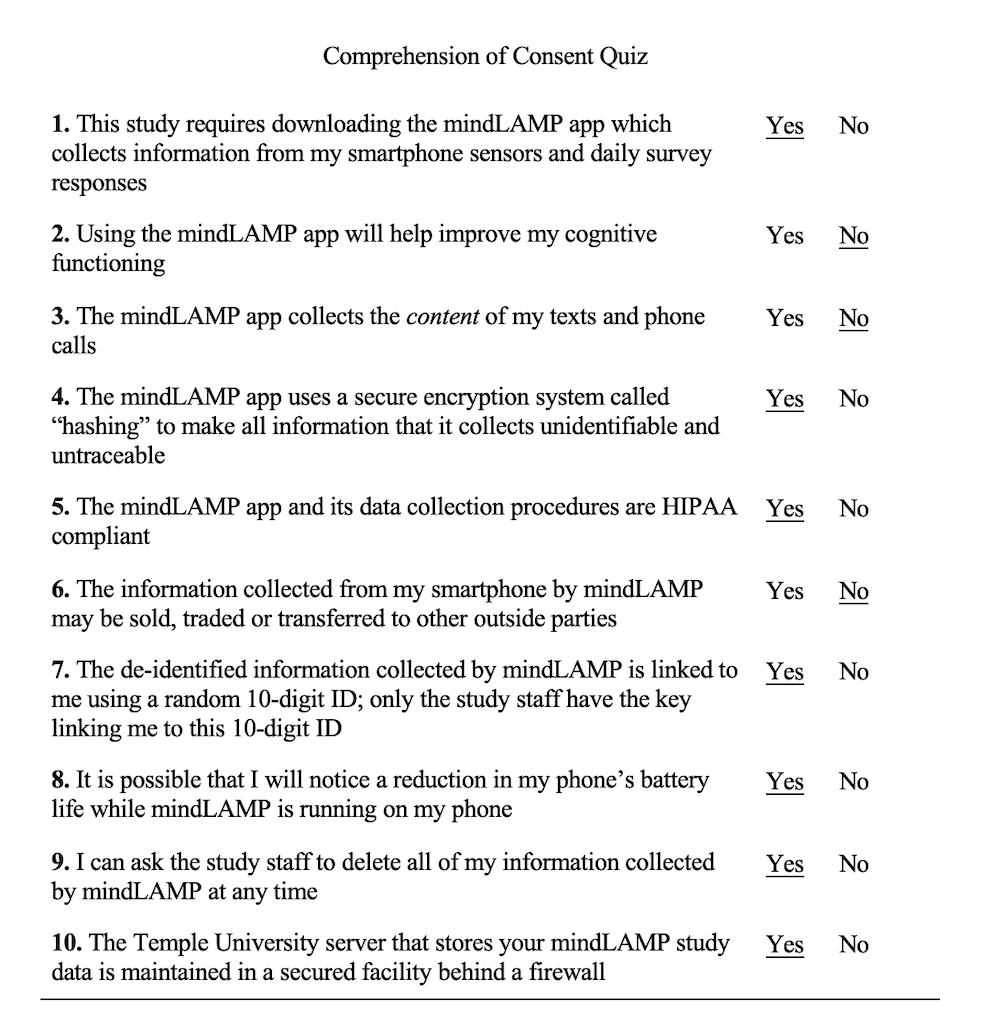

Supplement: Multimedia Appendix 1 [file humanfactors_v11i1e59974_app1.png]
